# Delay in tuberculosis diagnosis and treatment in Amhara state, Ethiopia

**DOI:** 10.1186/s12913-019-4056-7

**Published:** 2019-04-16

**Authors:** Melashu Balew Shiferaw, Amtatachew Moges Zegeye

**Affiliations:** 1 Amhara Public Health Institute, P.O.Box 447, Bahir Dar, Amhara Ethiopia; 2 North Shoa Zone Health Department Tuberculosis, HIV and Leprosy program coordinator, Debre Birhan, Shoa Ethiopia

**Keywords:** Tuberculosis, Delay, Diagnosis, Health information

## Abstract

**Background:**

Delayed presentation is a major problem contributing to the high burden and transmission of tuberculosis (TB) in developing countries. The delay may be due to patient delay if the patient visits health-facility for diagnosis after the onset of symptoms of more than 3 weeks or health system delay if the patient is not diagnosed and treated at the time of the first visit. Ethiopia, where no more than two-thirds of TB cases are detected is no exception. Therefore, the aim of this study was to assess delay in diagnosis of tuberculosis among patients taking anti-TB treatment in North Shoa Zone, Ethiopia.

**Methods:**

Institution based cross-sectional study was conducted from 01 to 30 December 2017. All TB patients who took their treatment in the health facilities of the seven selected districts of North Shoa Zone were included. Data was entered into EPI INFO version 3.5.1 statistical software and transferred into SPSS version 20.0 for further analysis. Bivariate and multivariate analysis was used to identify associated factors for delayed TB diagnosis.

**Results:**

Out of 170 tuberculosis patients included, 162 patients were studied with a response rate of 95.3%. The proportion of tuberculosis patients who had delayed diagnosis was 59.9%. The mean time of health-seeking after developing the symptom of tuberculosis was 7.6 weeks. Tuberculosis patients with extra pulmonary site involvements were about four times more likely to be delayed in seeking health services (OR: 4.00, 95% CI: 1.77–9.03) as compared to patients with pulmonary TB. New patients were about three times more likely to come lately for TB diagnosis (OR: 2.94, 95% CI: 1.26–6.84) as compared to patients who had previous-history of treatment. Patients who had no information about TB before they started TB treatment were also around three times to be delayed (OR: 3.37, 95% CI: 1.43–8.00) as compared to those who had the information.

**Conclusions:**

More than 50% of TB patients reported in health-seeking relatively a longer time. Strengthening the health education activities for the community about tuberculosis and capacity building of the health care provider to increase suspicion of identifying tuberculosis and early diagnosis is crucial.

**Electronic supplementary material:**

The online version of this article (10.1186/s12913-019-4056-7) contains supplementary material, which is available to authorized users.

## Background

Tuberculosis (TB) remains a major global health problem. It causes ill-health about 10 million people each year and is one of the top ten causes of death world-wide that kills three people within a minute [1–4]. For the past 5 years, it has been the leading cause of death from a single infectious agent. However, most people who develop TB disease can be cured with a timely diagnosis and correct treatment [4].

Ethiopia is still one among the 30 high burdens TB countries and The TB case detection rate is very low compared to the World Health Organization (WHO) target of detecting all infectious TB cases to reach annual decline of TB incidence by 4.5 to 5% [3–6].

If not totally missed, delayed identification and diagnosis of TB cases plays a vital role in the transmission of the disease in the community in most developing countries. The delay may be due to patient delay if the patient visits health-facility for diagnosis after the onset of symptoms of more than 3 weeks or health system delay if the patient is not diagnosed and treated at the time of the first visit. There is also delay from diagnosis to treatment initiation if treatment is not stated as soon as the diagnosis is done. Ethiopia, where not more than two-thirds of all cases are detected is not exceptional. Delay in the diagnosis of TB exists between recognition of symptoms and initiation of treatment [5] and late diagnosis of tuberculosis is likely to be associated with a worse prognosis owing to the presence of extensive disease and poor clinical condition [7–12]. According to WHO, case fatality rate (CFR) reduction has been targeted to be 10% and lower. However, the CFR was 26% in Ethiopia in 2017, which is very far from the target and more than half of the cases were due to delay in diagnosis and treatment [4].

Many people with active tuberculosis do not experience typical symptoms in the first stages of the disease and may not seek care early and tested for TB. To cure TB and cut disease transmission, patients should be diagnosed early and be placed on effective treatment soon after diagnosis [13].

In this regard, health facility-based studies have explored the duration of delay and factors related to health seeking among pulmonary patients. A study conducted in Ethiopia showed that there was health seeking behavior of TB suspects and no diagnostic capacity of a health facility in all forms of TB [14]. As these groups of cases may need well-organized health facility which may be more inaccessible, so these patients may long last without the diagnosis. In different localities and different geographical areas, information on diagnostic delay and contributing factors is thus important for the evaluation and improvement of TB control programs. Therefore, the aim of this study was to assess delay in TB diagnosis among patients taking TB treatment at the health facility level in North Shoa Zone, Ethiopia.

## Methods

### Study design and period

Institution based cross-sectional study design was conducted from 01 to 30 December 2017 to assess the delay in diagnosis of TB and associated factors for patients who were taking TB treatment at health centers in North Shoa zone, Ethiopia.

### Study site and population

The study was conducted in seven districts of North Shoa Zone. North Shoa is one of the 11 Zones in the Amhara regional state. According to the central statistical agency estimate, the total population of the zone was 2,202,023. The zone has 24 administrative districts, 9 hospitals, 95 health centers and 389 health posts. TB is a major public health problem in the zone. According to the zonal health office report, there were 1973 new and 126 previously treated TB patients in 2017 alone. Health posts refer TB suspects to health centers for diagnosis of TB, whereas, hospitals and health centers provided TB diagnosis and treatment. As part of the TB prevention and control program, the health centers and hospitals should diagnose TB suspects and treat them immediately to reduce burden of TB as delayed diagnosis could lead to increased TB transmission especially in the era of MDR TB. Hence, this study was designed to assess whether there was a delay or not in the setting and share the information to take corrective actions for the problem of TB burden in the zone. Patients who took anti-TB treatment in all government health facilities in the sampled districts of North Shoa zone was the study population.

### Sample size

The sample size was calculated using a single population proportion formula based on the following assumptions (N = Ž _½_ P1 (P1-Q2)/W^2^):

Proportion of delay in seeking health care after the onset of symptoms of TB was taken 89.9% (study from Bale zone southeast Ethiopia [14]), significant level at α = 0.05, at 95% confidence interval, margin of error was 5 and 10% non-response rate, the minimum sample size calculated was 143 TB patients. But to increase the sample size for higher precision in estimate, all TB patients who were taking TB treatment in all health facilities of selected districts at the time of data collection were included in the study regardless of age and sex. Only one TB patient who was unable to respond due to serious health conditions was excluded from the study.

### Sampling procedure

**Fig. 1:**
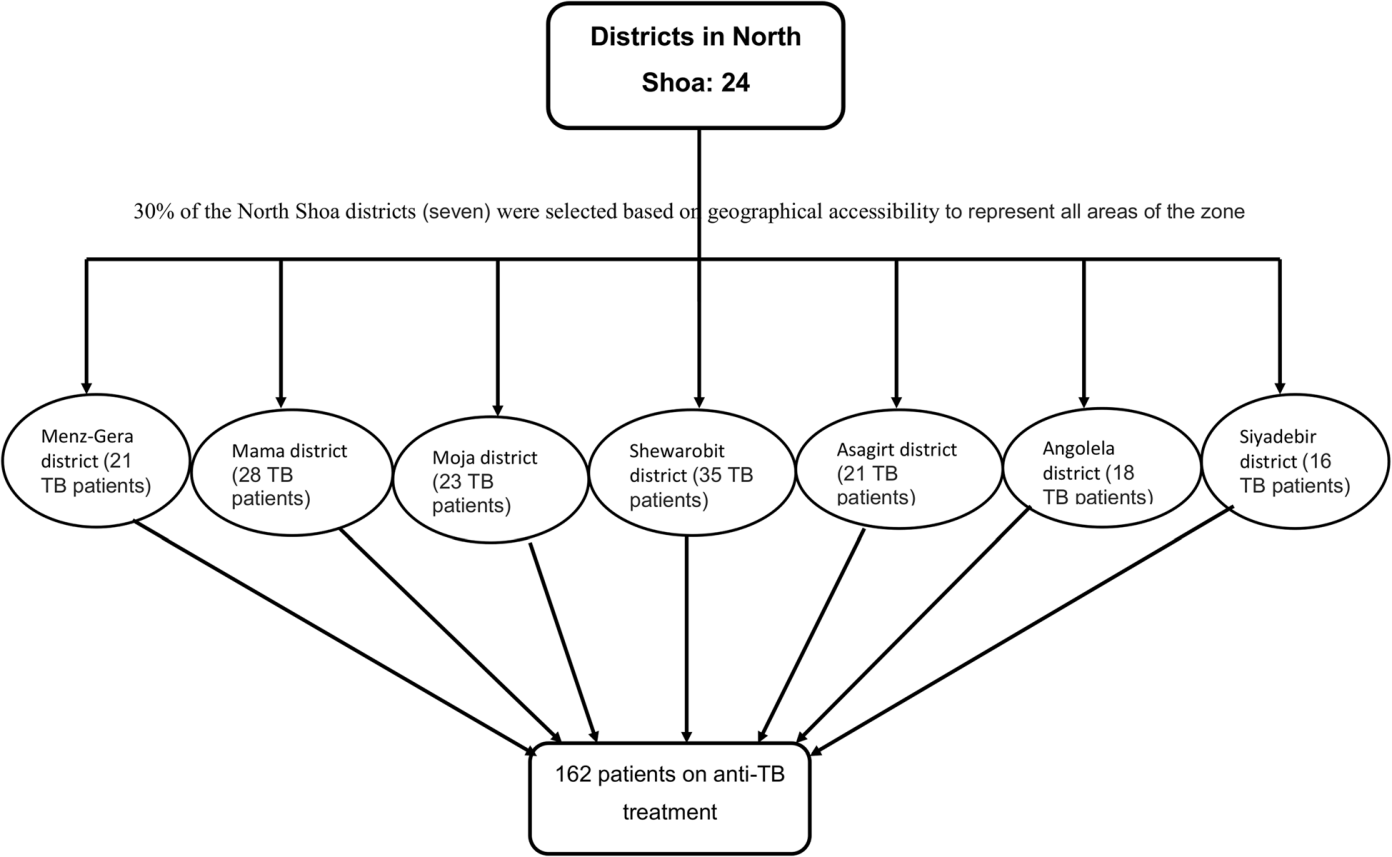
Sampling procedure of study subjects for the identification of delayed TB diagnosis

There were a total of 24 districts in the north Shoa zone. About 30% of the total districts (30% of 24 = 7.2 ≈ 7) were selected based on their cluster grouped using geographical proximity /remote or near to the capital of the zone/ and climate of the districts found in the zone (hot or cold areas) in order to represent the whole part of the North Shoa zone. Even though the sample size calculated was 143, all the patients who took anti-TB treatment in the government health facilities of the seven selected districts were included in the study at the time of data collection to have higher precision in estimate by increasing the sample size. Finally, a total of 162 patients under TB treatment in the seven districts were included consecutively (Fig. 1) when they came for collection of drugs.

### Operational definition

#### Patient delay

Is defined as the period between the onset of the illness and patient first attendance of health care facility because of this illness. The patient was said delayed if he/she visited health-facility after onset of symptoms for more than 3 weeks (2 week entry point for presumptive Tb case and 1 week period to seek health care) [15].

#### Health system delay

Is the period between patient’s first attendance of health care facility with symptoms of TB and the diagnosis of Tuberculosis. The patient was said delayed if TB is not diagnosed and treated at the time of first visit [15].

The date of onset for the main symptoms was taken as the date of onset for the illness. For PTB patients, cough was taken as the main symptom whereas for EPTB patients, either localizing symptoms like swelling for TB lymphadenitis, chest pain for TB pleurisy or constitutional symptoms (fever, night sweats, weight loss, and loss of appetite) were taken as the onset of the illness whichever came first [15].

### Data collection procedure

Data collection tool was first prepared in English and then translated to Amharic (local language). The second version of the tool was retranslated into the original one by language experts and investigators to check its consistency. The edited final version of the questionnaire was used to collect data from study subjects and from parents/guardians for children.

Trained district health office TB officers, one for each district, collected the data. Interview technique was used to collect data with structured and pre-tested questionnaire containing socio-demographic and treatment profile of TB patients. Data quality control pretest was done at Ataye health center. The health center that was not included in the study. The questionnaire was checked for consistency and completeness before conducting real data collection (see Additional file 1). Training was given for data collectors and supervisors about research objectives, data collection tools, procedures and interview techniques for 1 day. The data were collected by interviewing the study participants. However, data from children were collected using interview of the parents/guardians. The investigators, together with five supervisors supervised the data collection and checked completeness of the entire returned questionnaires daily.

### Data processing and analysis

The returned data we**r**e entered into EPI info version 3.5.1 and transferred to Statistical Package for Social Science (SPSS) version 20.0 software for analysis. Descriptive and summary statistics were calculated. The patient was said delayed if he/she visited health facility for diagnosis after onset of symptoms more than 3 weeks after the onset of symptoms. Health system delay was considered if the patient was not diagnosed at the time of first-visit. Bivariate and multivariate logistic regression was used to identify associated variables. The multivariate logistic regression was adjusted using backward LR method. Variable having P- value up to 0.2 in bivariate analyses was entered into multivariate model. Variables with *P*-value < 0.05 in the multivariate analysis was considered as significantly associated with delay in TB diagnosis.

### Ethical considerations

Ethical clearance was assured prior to data collection from the Amhara Public Health institute Ethical Review. Permission was obtained first from North Shoal Health Department, then from the administrators of all health institutions. Each study participant was informed about the purpose, methods of collection, benefits and risk of the study by the data collector. Written informed consent was obtained from each participant. Regarding children the consent was found from their parents/guardians. They were informed that they had the right to refuse or withdraw from the interview and it would not have any effect on the services they would have.

## Results

### Socio-demographic characteristics of study participants

Out of 170 tuberculosis patients included in this study, 162 (95.3%) responded to participate in the study. The mean age of the respondent was 33.60 (± 1.47 years). Ninety-two (56.8%) respondent were male and 51.2% of the respondents were from rural area. Majority of the respondents (89.5%) were orthodox Christian religion followers. Out of the total participants, 72 (44.2%) were married and 60 (37%) could not read and write (Table 1).

**Table 1:** Characteristics of tuberculosis patients in seven districts of North Shoa zone, Ethiopia, December 2017

Variable	Number	Percent (%)
Sex		
Male	92	56.8
Female	70	43.2
Age in years		
1–10	5	3.1
11–20	29	17.9
21–29	49	30.2
30–39	32	19.8
40–50	29	17.9
> 50	18	11.1
Marital status		
Married	72	44.4
Not married	65	40.1
Divorced	12	7.4
Widowed	13	8
Resident		
Rural	83	51.2
Urban	79	48.8
Religion		
Orthodox	145	89.5
Muslim	16	9.9
Protestant	1	0.6
Educational status		
Cannot read and write	60	37
Can read and write	56	34.6
Primary	34	21
Secondary	12	7.4
Number of Family		
0–2	39	24.1
3–6	111	68.5
7–9	12	7.4
Jobs of respondent		
Government employee	17	10.4
Self employee	44	27.1
Housewife	2	1.2
Farmer	74	45.6
Student	16	9.9
Other	9	5.8
Distance travel		
0-5 km	91	56.2
Greater than 5 km	71	43.8

### Prevalence of delay and other variables of tuberculosis patients

Table 2 describes the prevalence of delay and other related parameters of tuberculosis patients in the settings. The proportion of tuberculosis patients who had early health seeking (within 21 days of onset of the symptom) was only 40.1%. The rest 59.9% of patients visited lately. Mean time of health seeking after developing the symptom of tuberculosis was 7.6 weeks (± 1.22 weeks).

**Table 2:** Care seeking and investigations among tuberculosis patients in seven districts of North Shoa zone, Ethiopia, December 2017

Variable	Number	Percent
How many times you go to health facility before you disease has been diagnosed?		
1	30	18.5
2	58	35.8
3	28	17.3
4	13	8.1
5	17	10.5
6	5	3.1
7	5	3.1
8	4	2.4
12	2	1.2
Delayed health seeking		
Diagnosed at first visit	65	40.1
Not diagnosed at first visit	97	59.9
Major reasons for patient delay for more that 21 days		
Symptom disappear by itself	76	79
Financial constrain	10	10
Used herbal medicine	4	4
Being HF distant	7	7
Category of TB		
New	140	86.4
previously treated	22	13.6
The perception of cause of TB by the patients		
Due to draft	78	48.2
Bacterial infection	59	36.4
Dust particle	14	8.6
Others	11	6.8
Where do you go first when you develop sign of TB		
Health post	45	27.7
Hospital	59	36.4
Private HF	44	27.2
Holly water	9	5.6
Other	5	3.1
Asked about TB at first visit		
yes	92	56.8
No	70	43.2
Type of TB		
Pulmonary	115	71
Extra pulmonary	47	29
Give Sputum Sample at first visit		
Yes	86	53.1
No	76	46.9
Previous contact history		
Yes	43	26.5
No	119	73.5
Who initiated you to Health facility		
The patient by itself	75	46.3
Spouse of the patients	22	13.6
Health extension worker	10	6.1
My family	19	11.7
Other relative	24	14.9
HAD leaders	6	3.7
Other	6	3.7

While the range of coming to the health-facility after developing the symptom was early as 1 week to 103 weeks. On the other hand, the mean of the second delay (after a patient went to the health facility to start TB treatment) was 33.3 days and ranges as early as 1 week to 51 weeks. Until the disease diagnosed, the patients had repeatedly come to the health-facility on average 3 (± 1.9) times.

### Factors associated with delay in health seeking of tuberculosis patients

Result from multivariate analysis showed that clients with pulmonary tuberculosis, who had got information about tuberculosis and those with the history of previous TB treatment were significantly and independently associated with delay in health seeking. Sex of respondent, age, educational status, residence, marital status, type of health-facility first attended, history of previous contact, distance travel and religion of the respondent were not significantly associated with delay in health seeking of tuberculosis.

Accordingly, tuberculosis patients with extra pulmonary site involvements were about four times more likely to delay in seeking health service (OR: 4.00, 95% CI: 1.77–9.03) as compared with patients with only pulmonary site involvement. New patients were about three times more likely to come lately for TB diagnosis (OR: 2.94, 95% CI: 1.26–6.84) as compared with those who had previous-history of treatment. Tuberculosis patients who had no information about tuberculosis in before they started this treatment were also around three times more likely to delay in health service seeking for tuberculosis treatment (OR: 3.37, 95% CI: 1.43–8.00) as compared with those who had any information about tuberculosis before they started the treatment (Table 3).

**Table 3:** Determinants of delay in diagnosis of tuberculosis in seven districts North Shoa zone, Ethiopia, December 2017

Variable	Delay in health seeking		Crude OR (95%CI)	Adjusted OR (95%CI)
	Yes n (%)	No n (%)		
Type of Tb (in site)				
Pulmonary	59 (36.4)	56 (34.6)	1.00	1,00
Extra pulmonary	38 (23.5)	9 (5.6)	4.0 (1.77–9.03)	3.41(1.46–7.95)
Treatment history of patients				
New	91(56.2)	49 (30.2)	4.9 (1.8–13.4)	2.94 (1.26–6.84)
Previously treated	6 (3.7)	16 (9.6)	1.00	1.00
Heard information about TB				
Yes	63 (38.9)	56 (34.6)	1.00	1.00
No	34 (21)	9 (5.6)	3.36(1.48–7.61)	3.37 (1.43–8.00)

OR was adjusted using multivariate logistic regression backward LR method

## Discussion

Delay in health-seeking of tuberculosis patients have a number of public health impacts in the patients as well as in the community and country as a whole like unfavorable treatment outcome, increase transmission of the disease in the community and economic crisis in the country. An important preventable period of infectiousness in the community may be increased due to the habit of life priority and low awareness as well as once they came to the health-facility very low suspicion and identification of presumptive TB case in the health service provision [3, 4].

The delay in TB diagnosis found in this study (59.9%) is higher than the study done in east Wollega (42.3%), Ethiopia [9]. It may be due to variation in study subjects, where only pulmonary TB cases were included in Wollega. When we compare it with the country goal of ending TB strategy, it is far from the target because more than half of TB patients were delayed that could spread TB in the community. Out of those who delayed for health seeking, 79% of them believe that they will get relief by itself. This may indicate the low community awareness and miss conception about the disease provided that any presumptive TB case has to undergo proper evaluation for TB. Moreover, 48% of study participants believed that the case of TB is due to draft. This may indicate that the general lack of awareness about the source of TB infection so that they were not able to prevent the disease.

Beyond the delay in health seeking at community level, only 56% of TB patients were asked about symptoms of TB at first health facility visit and 53.4% gave sputum samples for AFB diagnosis. This indicates that there were miss opportunities in addressing the TB patients and regarding performing sputum AFB, it may be due to the low accessibility of the service as only 60% of the health facilities had the service in north Shoa zone districts. This problem was also seen in a Taiwan study that showed increased health system delay to diagnose and treat tuberculosis between 2003 and 2008 [16]. On the other hand, 27.8% of patients had first-visit at health post, which is higher compared to studies done in Wollega (17%) and Bale regions (14.8%) [9, 14] even though it did not show significant difference.

The result of this study showed that the site of TB disease is a factor for delay in health seeking. Accordingly, TB patients with extra pulmonary site involvements were about four times more likely to delay in seeking health service when developing signs and symptoms of TB as compared with patients with only pulmonary site involvement. This is similar with a study done in Bale [14] where extra pulmonary patients were delayed than that of pulmonary patients. This may be due to the fact that the signs and symptoms of extra pulmonary TB patients may be more constitutional and organ-specific so that it may be difficult to recognize early by the community as pulmonary cases. Although EPTB patients may not be more infectious to the community, they may suffer long-term disease, and disability.

This study found that new TB patients were about three times more likely to come lately for TB diagnosis as compared with those who had previous history of anti-tuberculosis treatment. It is similar with a study done in the rural part of Ethiopia [17]. This may indicate that once patients develop the disease, they will get information about the disease and for the next time when they develop sign and symptoms, they will seek treatment early.

Tuberculosis patients who did not have information about TB were more likely to delay in health-seeking. Accordingly, tuberculosis patients who had no information about tuberculosis in different way before they started this treatment were also around three times more likely to delay in health service seeking for tuberculosis as compared with those who have any information about tuberculosis. This finding is similar with a study conducted about knowledge and health seeking behavior of TB patients in south-west Ethiopia [17]. This may indicate that there were no clear information and awareness creation activities in the community.

Hence, the role of health facilities, districts and the zone health department is tremendous and needs to plan, coordinate, and evaluate TB control and prevention efforts. As part of the prevention and control program early TB diagnosis is one of the strategies in order to reduce the spread of TB in the region. In addition, district health office and the zonal health departments should focus and provide oversight the clinical and diagnostic services for patients with TB and their contacts, training and education, and monitoring and evaluation to solve the problem further.

As limitation, the study participants were selected after the TB diagnosis that may lead to a recall bias. In addition, underlying diseases of the patients’ status such as diabetes mellitus and HIV status were not considered during the time of data collection.

## Conclusions

This study showed that almost 60% of TB patients were reported health seeking relatively in longer time. There was also delayed diagnosis at health facility level after the patients had initiated health seeking despite different effort made to increase the index of suspicion and capacity of health care worker and health facility to end the TB epidemic. Lack of information about tuberculosis, previous treatment history, and site of the disease were factors for delayed diagnosis. Strengthening community health education activities about tuberculosis and capacity building of the health care providers are important interventions to increase suspicion of identifying tuberculosis and importance of early diagnosis.

## Additional file

Additional file 1: Interview guide questionnaire. (DOCX 18 kb)

## Abbreviations

EPTB: Extra pulmonary tuberculosis
PTB: Pulmonary tuberculosis
TB: Tuberculosis
WHO: World Health Organization
