# Supplementary material for: Delay in tuberculosis diagnosis and treatment in Amhara state, Ethiopia
Source: BMC Health Serv Res. 2019 Apr 16;19:232. doi: 10.1186/s12913-019-4056-7 (PMC6469046; doi:10.1186/s12913-019-4056-7)
Supplement: Supplementary file 1 — Interview guide questionnaire. (DOCX 18 kb) [file 12913_2019_4056_MOESM1_ESM.docx]

Questionnaire

| S,N | List of questions | List of answers | | Go to | |
| --- | --- | --- | --- | --- | --- |
| 01 | Age | __________ years | |  | |
|  | Sex | 1. Male 2. female | |  | |
| 02 | Residence | 1.rural  2.urban | |  | |
| 03 | Marital status | 1. married  2. not married  3. Divorced  4. Widowed | |  | |
| 04 | Religion | 1. Orthodox Christian  2. Muslim  3. Protestant  4. Others | |  | |
| 05 | Educational status | 1. can’t read and write  2. primary school (1-8)  3. Secondary school(9-12)  4. 12+ | |  | |
| 06 | Monthly income | ------- Ethiopian birr in year | |  | |
|  | Number of family in the household |  | |  | |
| 07 | jobs | 1. government employee  2. self-employee  3. housewife  4. farmer  5+. others | |  | |
| 8 | Distance from health facility | --------------------- km | |  | |
|  |  | | | | |
| 9 | Type of TB | | 1. Pulmonary TB  2 Extra pulmonary TB | |  |
| 10 | Category of TB | | 1. New 2. Previously treated | |  |
| 11 | What do you think is the cause of TB | | 1. Due to draft 2. Due to bacterial 3. Due to dust 4. Others | |  |
| 12 | Do you know how TB is transmitted? | |  | |  |
| 13 | What is sign and symptoms of TB  (more than one answer is possible) | | 1.cough of two weeks and above  2.,Night sweating  3. loss of appetite  4.weight loss  5. enlargement of lymph nods  6. others | |  |
| 14 | Which sign and symptoms do you face before starting treatment | |  | |  |
|  |  | |  | |  |
| 15 | Where do you go first when you see the sign and symptoms diseases | | 1. To HP 2. To HC /Hospitals 3. To private health institution 4. To holly water 5. Tenkuay/awaka 6. other | |  |
| 16 | After what days of the sign and symptom of TB you go to HF | | --------- day after | |  |
| 17 | If you delayed more than 21 day, what was the reason? | | 1. Assuming I will vet relief by itself 2. HF is distant from me 3. I have used alternative medication 4. I have no money | |  |
| 18 | How many times go to HF before it diagnosed as TB | |  | |  |
| 19 | How many days you lasted between your first visit and initiation of treatment | |  | |  |
| 19 | Have you asked about the sign and symptoms of TB at your first visit of HF | | 1. Yes 2. No | |  |
| 20 | If yes, have you ordered to give sputum for AFB? | | 1. Yes 2. No | |  |
| 21 | If AFB was not done, what kind of medication you got | | 1. I have given medication 2. Other lab test is done | |  |
| 22 | Who initiate you to go to health facility? | | 1. Me myself 2. My husband/wife 3. My mother/father 4. Other relatives 5. HEW 6. HAD leaders 7. others | |  |
| 23 | Before you start your treatment, have any close contact with TB patients | | 1. yes 2. No I do not have any contact | |  |
| 24 | Before you start your treatment, have you heard about the TB | | 1. Yes 2. No I do not heard | |  |
| 25 | If yes for the above question, from whom do you get the information | | 1. From health center/hospital staff 2. From HEW 3. Health development army | |  |

Name of the data collectors___________________ signature

Name of HF ______________________

Date ______________________

Name of the supervisor ------------------ ------- signature
